# Association between *MTHFR* C677T and A1298C gene polymorphisms and maternal risk for Down syndrome: A protocol for systematic review and/or meta-analysis

**DOI:** 10.1097/MD.0000000000028293

**Published:** 2022-01-21

**Authors:** Carla Talita Azevedo Ginani, Jefferson Romáryo Duarte da Luz, Saulo Victor e Silva, Fabio Coppedè, Maria das Graças Almeida

**Affiliations:** a Post-graduation Program in Health Sciences, Federal University of Rio Grande do Norte, Health Sciences Center, Natal, Rio Grande do Norte, Brazil; b Multidisciplinary Research Laboratory, DACT, Health Sciences Center, Federal University of Rio Grande do Norte, Natal, Brazil; c Post-graduation Program in Pharmaceutical Sciences, Health Sciences Center, Federal University of Rio Grande do Norte, Natal/RN, Brazil; d Department of Translational Research and of New Surgical and Medical Technologies, University of Pisa, Pisa, Italy; e Interdepartmental Research Center Nutrafood “Nutraceuticals and Food for Health”, University of Pisa, Pisa, Italy.

**Keywords:** case–control studies, Down syndrome, gene polymorphisms, maternal risk, meta-analyses

## Abstract

**Background:**

Down syndrome (DS) is one of the most common chromosomal abnormalities among live-born babies and one of the best-known intellectual disability disorders in humans. Errors leading to trisomy 21 are primarily arising from defects in chromosomal segregation during maternal meiosis (about 88% of cases), and the focus of many investigations has been to identify maternal risk factors favoring chromosome 21 malsegregation during oogenesis. Maternal polymorphisms of genes required for folate metabolism are the most investigated risk factors for the birth of children with DS. Through this review, we sought to investigate the association of the polymorphisms “C677T” and “A1298C” of the *MTHFR* gene with maternal risk for DS.

**Methods:**

We will use the databases PubMed, Embase, Scopus and Web of Science to search for case-control studies published from 1999 up to September 2021 without language restriction. Results will be presented as relative risks and 95% confidence intervals for dichotomous outcomes and mean differences, or standardized mean differences along with 95% confidence intervals, for continuous outcomes. The all data synthesis will be analyzed on the Review Manager 5.2 version software.

**Results:**

This study will be able to clarify all the doubts we seek and that it will be able to provide accurate data that will be able to describe how these polymorphisms can act to increase the predisposition for the birth of children with DS in different populations and under different dietary conditions.

**Conclusions:**

This study will clarify the relationship between C677T and A1298C polymorphisms *MTHFR* gene with increased the maternal risk for Down syndrome.

**Registration:**

This systematic review and meta-analysis protocol has been registered on the Prospective Registry of International Systematic Review and Meta-analyses: CRD42021269338.

## 1 Introduction

Down syndrome (DS) is one of the most common chromosomal abnormalities among live-born babies and one of the best-known intellectual disability disorders in humans.^[1,2]^ The disease results from the presence of an extra copy of the genetic material of chromosome 21, and in most cases (95%) from a total trisomy of chromosome 21.^[1]^

Errors leading to trisomy 21^[1,2]^ are primarily arising from defects in chromosomal segregation during maternal meiosis (about 88% of cases), and the focus of many investigations has been to identify maternal risk factors favoring chromosome 21 malsegregation during oogenesis.^[3,4]^ Although the only well-established risk factor for DS is still advanced maternal age, the birth of children with this syndrome from women under 35 years is increasingly common, suggesting that other factors may influence chromosome 21 malsegregation.^[3,4]^

Folate metabolism provides one-carbon units required for DNA synthesis and methylation, thus playing a pivotal role in maintaining genome stability during cell divisions.^[5]^ In 1999, James and coworkers hypothesized that an abnormal maternal folate metabolism resulting from common polymorphisms in genes involved in the metabolism of this nutrient, could impair the methylation levels of chromosome 21 pericentromeric regions, favoring its meiotic non-disjunction.^[6]^ Several subsequent studies carried out for more than 15 years support a potential contribution of polymorphisms of genes required for folate metabolism as maternal risk factors for the birth of a child with DS.^[7]^

Methylenetetrahydrofolate reductase (*MTHFR*) is one of the most important enzymes in folate metabolism.^[8]^ It converts 5,10-methylenetetrahydrofolate into 5-methyltetrahydrofolate, an active form of folic acid acting as methyl donor for the remethylation of homocysteine to methionine in a reaction catalyzed by methionine synthase. The methionine is then converted into *S*-adenosyl-methionine (SAM), which is the main intracellular methyl donor compound for methylation reactions.^[5,8]^

The most well known polymorphisms of the *MTHFR* gene, namely C677T and A1298C, are directly involved in a decrease of enzyme activity.^[9,10]^ The *MTHFR* C677T polymorphism has been the first polymorphism in genes coding folate metabolic enzymes to be associated with the maternal risk for having a DS child^[6]^ and represents the so far most investigated maternal genetic risk factor for trisomy 21.^[7]^ Particularly, the *MTHFR* C677T polymorphism (rs1801133) is the result of a transition mutation from C to T at nucleotide 677, causing an amino acid substitution (Ala222Val) in the catalytic domain of the protein.

The homozygous genotype (T/T) is responsible for the reduction in up to 70% in *MTHFR* enzymatic activity.^[9]^ The *MTHFR* A1298C polymorphism (rs1801131) is another common missense mutation leading to a Glu429Ala aminoacidic change in the regulatory domain of the protein and the homozygous genotype (C/C) results in about 40% reduction in enzymatic activity.^[10]^ Noteworthy, the *MTHFR* C677T and A1298C polymorphisms are in strong linkage disequilibrium (LD), and the 677T allele has been nearly always observed in *cis* with the 1298A one. LD is not complete, but very low frequencies have generally been reported for the rare 677T-1298C haplotype.^[11]^

A biological explanation for the LD existing between the two *MTHFR* polymorphisms was proposed considering the fact that *MTHFR* works as a dimer protein in which monomers associate head to tail, and the stability of the dimer depends on what aminoacid is present at position 222 and what at position 429, resulting from the combined *MTHFR* 677/1298 genotype.^[11]^ Consequently, the overall activity of the MTHFR protein is better explained by the combined *MTHFR* 677/1298 genotype, rather than by each single polymorphism alone.^[11]^

Physiological levels of folate stabilize the MTHFR dimer, but the *MTHFR* 677T allele renders the enzyme thermolabile, particularly in homozygous (T/T) individuals that are prone to dimer destabilization under conditions of reduced folate bioavailability.^[8]^ In summary, combinations of genetic and dietary factors contribute to MTHFR protein stability and activity.^[7]^ In addition, dietary folates and related metabolic factors can also regulate *MTHFR* gene methylation and expression levels.^[12,13]^

A recent study revealed that the fission yeast methylenetetrahydrofolate reductase protein (Met11) is important for maintaining the pericentromeric heterochromatin structure to ensure mitotic and meiotic chromosome segregation fidelity,^[14]^ supporting the original hypothesis by James et al and suggesting that *MTHFR* polymorphisms promote changes in global DNA methylation during maternal meiosis leading to chromosomal non-disjunction and increasing the maternal risk to have children with DS.^[6]^

In addition, both *MTHFR* C677T and A1298C polymorphisms have been linked to an increased risk of chromosome malsegregation in lymphocytes of mothers of DS children.^[15]^ Following the original study by James and coworkers,^[6]^ several investigators performed case–control studies to evaluate *MTHFR* C677T and/or A1298C polymorphisms as maternal risk factors for the birth of a child with DS, but the small sample-size of these studies coupled to differences in allele frequencies, lifestyles and dietary habits among different populations, yielded conflicting results.^[15–22]^

At least three different meta-analyses of those studies, performed between 2013 and 2014, addressed whether *MTHFR* C677T and/or A1298C polymorphisms are associated with the maternal risk for having a child with DS, and despite that significant associations emerged for the C677T one in the overall population, the different inclusion criteria selected by the authors yielded conflicting or inconclusive results after subgroup stratification according to ethnicity.^[23–25]^

More recent case–control studies have highlighted a different effect size of these polymorphisms in the maternal risk for a DS birth according to ethnicity, maternal age at conception, errors occurring at maternal meiosis I or II, gene-nutrient interactions, and combined *MTHFR* C677T/A1298C polymorphisms.^[7,26–28]^ Moreover, congenital heart defects (CHD) are frequently observed in DS babies and owing to the role of folate metabolism in cell division and methylation, there is increasing interest in searching whether maternal *MTHFR* polymorphisms could represent risk factors for CHD in DS newborns.^[7,17,21]^

What is missing is an updated systematic review and meta-analysis of the studies investigating *MTHFR* C677T and/or A1298C polymorphisms as maternal risk factors for DS addressing not only the overall contribution of each of the two polymorphisms to DS risk, but performed in such a way to extract data on combined genotypes, ethnicity, serum folate levels, maternal age at conception, errors occurred at maternal meiosis I or II, as well as data on CHD in the DS children, in order to shed light on the different role of the *MTHFR* genotype in the various subgroups. Therefore, the aim this review is to evaluate the association between frequency of the C677T and/or A1298C polymorphisms of the *MTHFR* gene with maternal risk for DS.

## 2 Objective

The aim of this systematic review and meta-analysis is to clarify the relationship between the C677T and A1298C polymorphisms *MTHFR* gene and the maternal risk for DS.

## 3 Methods

The elaboration of this study will be conducted according to Preferred Reporting Items for Systematic Review and Meta-analysis Protocol Guidelines.^[29]^ This protocol was registered in the prospective international registry of systematic reviews and the trail registration is number CRD42021269338 (https://www.crd.york.ac.uk/prospero/display_record.php?RecordID=269338).

### 3.1 Eligibility criteria

#### 3.1.1 Types of studies

Will be included studies have to meet the following criteria:

(1) articles should be published from 1999 up to September 2021 without language restriction;
(2) article should have sufficient data on both allele and genotype frequencies to calculate the odds ratio with 95% confidence intervals (CI);
(3) article should be case–control studies; and
(4) authors should describe the genotyping protocols.

Case–control studies not showing tabular data with *MTHFR* C677T and/or A1298C allele and genotype frequencies will be excluded as well as Review articles, editorials and/or commentaries, meeting abstracts and any other kind of article not published in a peer-reviewed journal.

#### 3.1.2 Participants

Studies shall include women who gave birth to at least one child with DS in this systematic review and meta-analyses. Women/mothers of children who do not have DS or other diagnosed syndromes, and without history of spontaneous miscarriages will be the control group.

#### 3.1.3 Primary outcome

Maternal risk for the birth of a child with DS.

#### 3.1.4 Secondary outcome

The role of polymorsphisms as risk factors for CHD in DS newborns.

### 3.2 Information sources

#### 3.2.1 Electronic searches

We will use four databases: PubMed, EMBASE, Scopus and Web of Science to search for articles of studies of case control published from 1999 up to September 2021 without language restriction.

#### 3.2.2 Search strategy

The search terms (MESH) that will be used to conduct the research are: (mothers OR pregnancy) AND (methylenetetrahydrofolate reductase OR MTHFR OR genetic polymorphism OR gene polymorphism) AND (down syndrome OR trisomy 21). The studies considered eligible will also be selected from the reference lists of the retrieved articles.

### 3.3 Data extraction and quality assessment

#### 3.3.1 Data extraction

**Figure 1:**
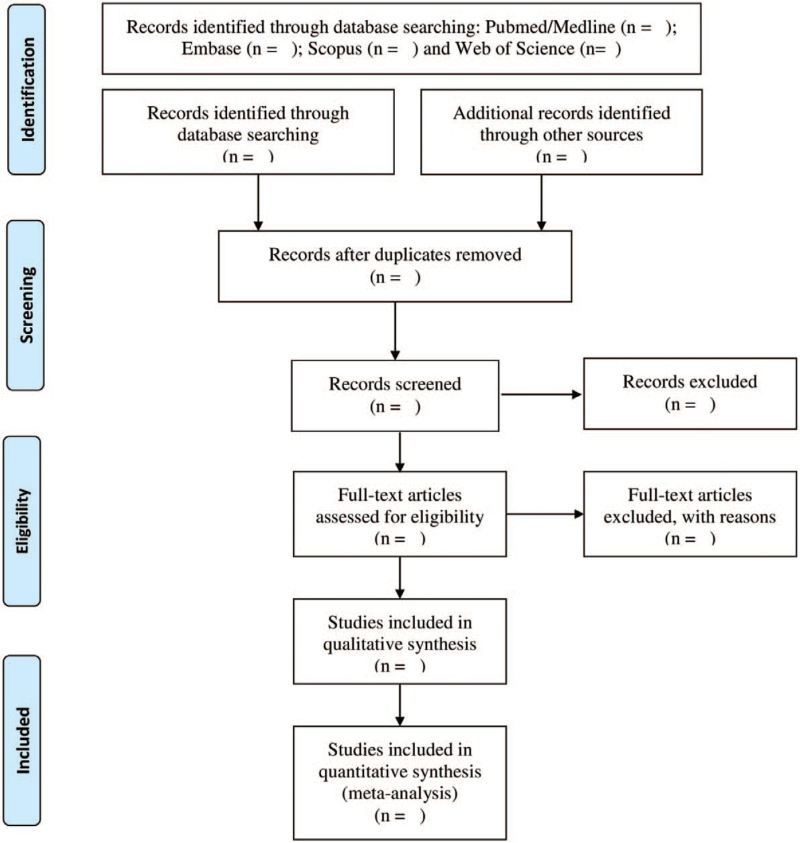
Flow diagram of the search for eligible studies for *MTHFR* gene polymorphism and maternal risk of Down syndrome: a systematic review and meta-analysis protocol (MTHFR = methylenetetrahydrofolate reductase).

Two authors, CTAG and JRDL, will independently examine the results of the research using the titles and abstracts. Duplicate studies and reviews will be excluded, and the studies by the same authors will be controlled for a possible overlapping of included patients. The reviewers will review the full text to determine whether the studies meet the inclusion criteria. Various characteristics of the eligible studies will be extracted: title, surnames of the first authors, year of publication, study location (country), study design, primary objective, population, sample size, follow-up period, inclusion/exclusion criteria, genotyping method, type of control and primary results. The process of selection of the studies is summarized in Figure 1.

#### 3.3.2 Risk of bias assessment

The assess the risk of bias in eligible studies using the Newcastle-Ottawa Scale 9-star tool. The quality of each study will be judged in three broad categories – namely: selection of the study population, comparability of the groups and determination of the exposure or the result of interest. The Newcastle-Ottawa Scale score vary from 0 to 9, in which (score 0–5) was low quality study and (score 6–9) was high quality study.^[30]^

### 3.4 Statistical analyses

The data will be entered into the Review Manager 5.2 version software (The Cochrane Collaboration, Software Update, Oxford, United Kingdom). For dichotomous results, the odds ratio (OR) and 95% CI for each study will be extracted or calculated. In case of heterogeneity (I^2^ ≥ 50%), the random effects model will be used to combine the studies to calculate the OR and 95% CI, using the DerSimonian-Laird algorithm in the meta for package, which provides functions to conduct meta-analysis in R. Other characteristics and results of the study will be summarized narratively if the meta-analysis cannot be performed for all or for some of the included studies. Sensitivity analyses will be used to explore the robustness of the results in relation to the quality of the study and the sample size. Sensitivity analyses will be shown in a summary table. If meta-analysis cannot be performed for all or some of the included studies, the results will be presented qualitatively.

### 3.5 Heterogeneity assessment

The heterogeneity between the results of the study will be assessed using a standard χ^2^ test with a significance level of *P* < .1. To assess heterogeneity, we plan to calculate the I^2^ statistic, which is a quantitative measure of inconsistency between studies. A value of 0% indicates no heterogeneity, whereas I^2^ values ≥50% indicate a substantial level of heterogeneity; however, heterogeneity will be assessed only if it is appropriate to conduct a meta-analysis.

### 3.6 Subgroup analyses

Will be perform subgroup analyses to investigate the effect of ethnic differences in allele frequencies, combined genotypes, circulating folate levels and maternal age at conception to analyze the type of meiotic error in modulating the maternal risk for DS resulting from *MTHFR* C677T and A1298C polymorphism. Standardized data extraction forms will be created specifically for this review and the results will be later inserted into a database. All data entries will be double rechecked.

### 3.7 Quality of evidence

The analysis of the evidence for all outcomes will be assessed using the Grading of Recommendations Assessment, Development and Evaluation Working Group methodology by classifying the evidence as high, moderate, low, or very low.^[31]^

### 3.8 Ethics and dissemination

Ethical approval is not necessary because it is a systematic review article that will use data published in the literature. The findings of this manuscript will be published in a peer-reviewed journal and will be updated if there is enough new evidence to alter our conclusions.

## 4 Discussion

Studies published since 1999 support a relationship between genetic polymorphisms of folate metabolic genes and the maternal risk for having a child with DS, albeit with varying effect size in different populations and under different nutrient bioavailability.^[7]^ Folate is an important nutrient required for DNA synthesis and methylation, and several in vitro studies have shown that its depletion causes chromosome damage and malsegregation in cultured cells.^[32,33]^ The folic acid, nothing else is a synthetic form of folate, a water-soluble vitamin whose active form is 5-methyltetrahydrofolate.^[29]^

Indeed, the rationale for the association between maternal polymorphisms of folate metabolic genes and chromosome 21 non-disjunction is an altered chromatin structure of chromosome 21 favoring its malsegregation during meiosis and resulting from the impaired folate metabolism caused by the polymorphisms.^[6]^ MTHFR is one of the major enzymes in folate metabolism, linking the folate and the methionine cycles, thus providing one-carbon moieties for DNA synthesis and methylation reactions.^[7]^

Common *MTHFR* polymorphisms, namely C677T and A1298C, impair MTHFR stability and activity overall leading to hyperhomocysteinemia and reduced SAM production, and have been largely investigated as maternal risk factors for spontaneous abortion and DS risk.^[6,7,16–28,34]^

Previous literature meta-analyses support an association between the maternal *MTHFR* C677T polymorphism and the risk of birth of a child with DS,^[23–25]^ but many questions are still unsolved. Particularly, it is becoming evident that combined *MTHFR* C677T/A1298C genotypes give rise to more or less stable MTHFR dimers, and that intracellular levels of folate, SAM, and metabolic cofactors regulate MTHFR protein stability and activity, as well as the promoter methylation and expression levels of the *MTHFR* gene itself,^[7,12,28]^ also the frequency of *MTHFR* polymorphisms and their combinations varies among different populations, likely as a result of complex gene-nutrient interactions over time that might have favored certain haplotypes.^[7]^

There is also evidence that genetic factors predisposing to chromosome 21 non-disjunction in maternal meiosis I might be different from those predisposing to errors occurring at maternal meiosis II.^[35]^ It is therefore time to perform an updated systematic review and meta-analysis of the literature concerning the association of the polymorphisms C677T and A1298C of the maternal *MTHFR* gene with DS risk in attempt to address all these points.

Studies conducted over the years have some limitations regarding the sample size of these studies, the differences in allele frequencies and how the lifestyle and eating habits of mothers of children with DS could influence the measurement of this risk. Furthermore, data resulting from the analysis of ethnicity in these groups, when not properly conducted, generate conflicting and inconclusive results.

With this protocol of systematic review and meta-analysis, we wish to identify the association of the polymorphisms C677T and A1298C of the MTHFR gene with the maternal risk for DS, as well as to perform subgroup analyses to investigate the effect of ethnic differences in allele frequencies, combined genotypes, circulating folate levels, maternal age at conception and type of meiotic error in modulating the maternal risk for DS resulting from these polymorphisms, and investigate their role as risk factors for CHD in DS newborns.

We hope that our study will be able to clarify all the doubts we seek and that it will be able to provide accurate data that will be able to describe how these polymorphisms can act to increase the predisposition for the birth of children with DS in different populations and under different dietary conditions. This could help to identify individuals and populations at higher risk and seek nutritional strategies that can be implemented to reduce the maternal risk for DS and associated congenital complications.

## Acknowledgments

We would like to thank to doctoral student, Medeiros, KS and Sarmento, ACA from Post-graduation Program in Health Sciences, University Federal of Rio Grande do Norte for the contribution for the preparing this protocol systematic review and meta-analysis.

## Author contributions

**Conceptualization:** Carla Talita Azevedo Ginani, Jefferson Romáryo Duarte da Luz, Saulo Victor e Silva.

**Methodology:** Carla Talita Azevedo Ginani, Jefferson Romáryo Duarte da Luz, Fabio Coppedè, Maria das Graças Almeida.

**Resources:** Carla Talita Azevedo Ginani, Jefferson Romáryo Duarte da Luz.

**Supervision:** Fabio Coppedè, Maria das Graças Almeida.

**Writing – original draft:** Carla Talita Azevedo Ginani, Jefferson Romáryo Duarte da Luz.

**Writing – review & editing:** Fabio Coppedè, Maria das Graças Almeida.

## References

[1] Antonarakis SE Skotko BG Rafii MS. Down syndrome. Nat Rev Dis Primers 2020;6:09. 10.1038/s41572-019-0143-7 PMC8428796 32029743

[2] Bull MJ. Down syndrome. N Engl J Med 2020;382:2344–52. 32521135 10.1056/NEJMra1706537

[3] Coppedè F. Risk factors for Down syndrome. Arch Toxicol 2016;904:2917–3729. 10.1007/s00204-016-1843-3 27600794

[4] Mikwar M MacFarlane AJ Marchetti F. Mechanisms of oocyte aneuploidy associated with advanced maternal age. Mutat Res 2020;785:108320. 10.1016/j.mrrev.2020.108320 32800274

[5] Coppedè F. The complex relationship between folate/homocysteine metabolism and risk of Down syndrome. Mutat Res 2009;682:54–70. 19524060 10.1016/j.mrrev.2009.06.001

[6] James SJ Pogribna M Pogribny IP. Abnormal folate metabolism and mutation in the methylenetetrahydrofolate reductase gene may be maternal risk factors for Down syndrome. Am J Clin Nutr 1999;70:495–501. 10500018 10.1093/ajcn/70.4.495

[7] Coppedè F. The genetics of folate metabolism and maternal risk of birth of a child with Down syndrome and associated congenital heart defects. Front Genet 2015;6:223. 26161087 10.3389/fgene.2015.00223 PMC4479818

[8] Martínez-Frías ML. The biochemical structure and function of methylenetetrahydrofolate reductase provide the rationale to interpret the epidemiological results on the risk for infants with Down syndrome. Am J Med Genet A 2008;146A:1477–82. 18446861 10.1002/ajmg.a.32308

[9] Ueland PM Hustad S Schneede J Refsum H Vollset SE. Biological and clinical implications of the MTHFR C677T polymorphism. Trends Pharmacol Sci 2001;22:195–201. 11282420 10.1016/s0165-6147(00)01675-8

[10] Weisberg I Tran P Christensen B Sibani S Rozen R. A second genetic polymorphism in methylenetetrahydrofolate reductase (MTHFR) associated with decreased enzyme activity. Mol Genet Metab 1998;64:169–72. 9719624 10.1006/mgme.1998.2714

[11] Ulvik A Ueland PM Fredriksen A. Functional inference of the methylenetetrahydrofolate reductase 677C>T and 1298A>C polymorphisms from a large-scale epidemiological study. Hum Genet 2007;121:57–64. 17115185 10.1007/s00439-006-0290-2

[12] Coppedè F Denaro M Tannorella P Migliore L. Increased MTHFR promoter methylation in mothers of Down syndrome individuals. Mutat Res – Fundam Mol Mech Mutagen 2016;787:01–6. 10.1016/j.mrfmmm.2016.02.008 26926955

[13] Grossi E Stoccoro A Tannorella P Migliore L Coppedè F. Artificial neural networks link one-carbon metabolism to gene-promoter methylation in Alzheimer's disease. J Alzheimers Dis 2016;53:1517–22. 27392858 10.3233/JAD-160210

[14] Lim KK Teo HY Tan YY. Fission yeast methylenetetrahydrofolate reductase ensures mitotic and meiotic chromosome segregation fidelity. Int J Mol Sci 2021;22:639. 10.3390/ijms22020639 PMC7827777 33440639

[15] Coppedè F Migheli F Bargagna S. Association of maternal polymorphisms in folate metabolizing genes with chromosome damage and risk of Down syndrome offspring. Neurosci Lett 2009;449:15–9. 18983896 10.1016/j.neulet.2008.10.074

[16] Hobbs CA Sherman SL Yi P. Polymorphisms in genes involved in folate metabolism as maternal risk factors for Down syndrome. Am J Hum Genet 2000;67:623–30. 10930360 10.1086/303055 PMC1287522

[17] Brandalize APC Bandinelli E Dos Santos PA Roisenberg I Schüler-Faccini L. Evaluation of C677T and A1298C polymorphisms of the MTHFR gene as maternal risk factors for Down syndrome and congenital heart defects. Am J Med Genet Part A 2009;149:2080–7. 10.1002/ajmg.a.32989 19725133

[18] Zampieri BL Biselli JM Goloni-Bertollo EM. Maternal risk for Down syndrome is modulated by genes involved in folate metabolism. Dis Markers 2012;32:73–81. 22377700 10.3233/DMA-2011-0869 PMC3826801

[19] Costa-Lima MA Amorim MR Orioli IM. Association of methylenetetrahydrofolate reductase gene 677C>T polymorphism and Down syndrome. Mol Biol Rep 2013;40:2115–25. 23184006 10.1007/s11033-012-2270-z

[20] Sukla KK Jaiswal SK Rai AK. Role of folate-homocysteine pathway gene polymorphisms and nutritional cofactors in Down syndrome: a triad study. Hum Reprod 2015;30:1982–93. 26040482 10.1093/humrep/dev126

[21] Babić Božović I Vraneković J Starčević Čizmarević N Mahulja-Stamenković V Prpić I Brajenović-Milić B. MTHFR C677T and A1298C polymorphisms as a risk factor for congenital heart defects in Down syndrome. Pediatr Int 2011;53:546–50. 21159028 10.1111/j.1442-200X.2010.03310.x

[22] Jackson RA Nguyen ML Barrett AN Tan YY Choolani MA Chen ES. Synthetic combinations of missense polymorphic genetic changes underlying Down syndrome susceptibility. Cell Mol Life Sci 2016;73:4001–17. 27245382 10.1007/s00018-016-2276-0 PMC11108497

[23] Wu X Wang X Chan Y Jia S Luo Y Tang W. Folate metabolism gene polymorphisms MTHFR C677T and A1298C and risk for Down syndrome offspring: a meta-analysis. Eur J Obstet Gynecol Reprod Biol 2013;167:154–9. 23295071 10.1016/j.ejogrb.2012.11.022

[24] Yang M Gong T Lin X. Maternal gene polymorphisms involved in folate metabolism and the risk of having a Down syndrome offspring: a meta-analysis. Mutagenesis 2013;28:661–71. 24068460 10.1093/mutage/get045

[25] Rai V Yadav U Kumar P Yadav SK Mishra OP. Maternal methylenetetrahydrofolate reductase C677T polymorphism and down syndrome risk: a meta-analysis from 34 studies. PLoS One 2014;9:01–15. 10.1371/journal.pone.0108552 PMC4180743 25265565

[26] Jaiswal SK Sukla KK Chauhan A Lakhotia AR Kumar A Rai AK. Choline metabolic pathway gene polymorphisms and risk for Down syndrome: an association study in a population with folate-homocysteine metabolic impairment. Eur J Clin Nutr 2017;71:45–50. 27677362 10.1038/ejcn.2016.190

[27] Jiajin L Shuyan C Ying W Junxiao C Xiudi W. Genetic polymorphisms in folate metabolism as risk for Down syndrome in the southern China. J Matern Fetal Neonatal Med 2019;32:2030–5. 29343135 10.1080/14767058.2018.1424818

[28] Vraneković J Babić Božović I Bilić Čače I Brajenović Milić B. Methylenetetrahydrofolate reductase Dimer configuration as a risk factor for maternal meiosis i-derived trisomy 21. Hum Hered 2020;85:61–5. 33784681 10.1159/000515121

[29] Shamseer L Moher D Clarke M. Preferred reporting items for systematic review and meta-analysis protocols (PRISMA-P) 2015: elaboration and explanation. BMJ 2015;350:g7647. 25555855 10.1136/bmj.g7647

[30] Wells GA Shea B O’Connell D. The Newcastle-Ottawa scale (NOS) for assessing the quality of nonrandomised studies in meta-analyses. http://www.ohri.ca/programs/clinical_epidemiology/oxford.asp.

[31] Guyatt GH Oxman AD Vist G. GRADE guidelines: 4. Rating the quality of evidence – Study limitations (risk of bias). J Clin Epidemiol 2011;64:407–15. 21247734 10.1016/j.jclinepi.2010.07.017

[32] Wang X Thomas P Xue J Fenech M. Folate deficiency induces aneuploidy in human lymphocytes in vitro-evidence using cytokinesis-blocked cells and probes specific for chromosomes 17 and 21. Mutat Res 2004;551:167–80. 15225591 10.1016/j.mrfmmm.2004.03.008

[33] Beetstra S Thomas P Salisbury C Turner J Fenech M. Folic acid deficiency increases chromosomal instability, chromosome 21 aneuploidy and sensitivity to radiation-induced micronuclei. Mutat Res 2005;578:317–26. 16005909 10.1016/j.mrfmmm.2005.05.012

[34] Hekmatdoost A Vahid F Yari Z. Methyltetrahydrofolate vs folic acid supplementation in idiopathic recurrent miscarriage with respect to methylenetetrahydrofolate reductase C677T and A1298C polymorphisms: a randomized controlled trial. PLoS One 2015;10:01–12. 10.1371/journal.pone.0143569 PMC4668025 26630680

[35] Chernus JM Allen EG Zeng Z. A candidate gene analysis and GWAS for genes associated with maternal nondisjunction of chromosome 21. PLoS Genet 2019;15:e1008414. 31830031 10.1371/journal.pgen.1008414 PMC6932832

